# Worsened arterial stiffness in high-risk cardiovascular patients with high habitual carbohydrate intake: a cross-sectional vascular function study

**DOI:** 10.1186/1471-2261-14-24

**Published:** 2014-02-21

**Authors:** Hiu-Ting Chan, Yap-Hang Chan, Kai Hang Yiu, Sheung-Wai Li, Sidney Tam, Chu-Pak Lau, Hung-Fat Tse

**Affiliations:** 1 Division of Cardiology, Department of Medicine, Queen Mary Hospital, the University of Hong Kong, Hong Kong, China; 2 Department of Medicine, Tung Wah Hospital, Hong Kong, China; 3 Department of Clinical Biochemistry Unit, Queen Mary Hospital, Hong Kong, China

**Keywords:** Macronutrient, Carbohydrate intake, Arterial stiffness, Pulse wave velocity, Secondary prevention

## Abstract

**Background:**

Previous studies suggested that high dietary carbohydrate intake is associated with increased cardiovascular risk through raised triglyceride and decreased high-density lipoprotein-cholesterol levels. However, the relation between carbohydrate intake and arterial stiffness has not been established. The purpose of this study was to examine this relation among high-risk cardiovascular patients.

**Methods:**

We studied the relation between dietary macronutrient intake and arterial stiffness in 364 patients with documented cardiovascular diseases or risk equivalent (coronary artery diseases 62%, ischemic stroke 13%, diabetes mellitus 55%) and in 93 age-and-sex matched control subjects. Dietary macronutrient intake was assessed using a validated food-frequency questionnaire (FFQ) for Chinese. Heart-ankle pulse wave velocity (PWV) was measured non-invasively with a Vascular Profiling System (VP2000, Colin Corp. USA). A dietary pattern with ≥60% total energy intake derived from carbohydrates was defined as a high-carbohydrate diet according to the Dietary Reference Intakes (DRI) for Chinese.

**Results:**

Subjects who consumed a high-carbohydrate diet had significantly higher mean PWV than those who did not consume a high-carbohydrate diet (*P = 0.039*). After adjustment for potential confounders, high-carbohydrate diet was associated with significantly increased PWV [B = 73.50 (10.81 to 136.19), *P = 0.022*]. However, there was no significant association between high-carbohydrate diet and PWV in controls (P = 0.634).

**Conclusions:**

High-carbohydrate diet is associated with increased arterial stiffness in patients with established cardiovascular disease or risk equivalent.

## Background

Epidemiological studies suggested that dietary macronutrients intake could modify the risk of cardiovascular disease (CVD) [1]. A dietary pattern with high carbohydrate intake from fruits, vegetables, whole grain cereals and low-fat dairy products, but low in saturated fat and total fat, also known as the Dietary Approaches to Stop Hypertension (DASH), has been shown to prevent CVD [2,3]. On the other hand, replacement of carbohydrate with protein or unsaturated fat in diet has also been suggested to reduce the risk of CVD [4]. Indeed, a lower percentage of energy intake from carbohydrate was associated with decreased blood pressure, plasma triglyceride and low-density lipoprotein cholesterol (LDL-C), and increased high-density lipoprotein cholesterol (HDL-C) [1,5,6]. Conversely, a higher carbohydrate intake may induce hypertriacylglycerolemia [7]^.^ In the Optimal Macronutrient Intake Trial to Prevent Heart Disease study, a low carbohydrate diet (partial replacement of carbohydrate with either protein or unsaturated fat) reduced blood pressure, decreased low-density lipoprotein cholesterol and heart disease risk in prehypertensive individuals [1,6]. In the Nurses’ Health Study, glycemic index was associated with lower triacylglycerol among healthy postmenopausal women [8]. In a study of healthy Korean adults, high carbohydrate intake (70% of energy intake) was associated with higher body-mass index (BMI), blood pressure, fasting glucose, triglycerides and LDL-cholesterol levels [9]. Furthermore, among insulin-resistant and obese persons, excessive intake of energy from carbohydrate >60% was associated with unfavorable changes in the lipid profile [6]. Even in patients with non-cardiometabolic chronic diseases, such as women previously treated for breast cancer, high carbohydrate intake was showed to increase fasting level of triacylglycerol [10].

Apart from its effects on the lipid profile, higher carbohydrate consumption also suppresses vascular endothelial function as demonstrated by reduced endothelium-dependent flow-mediated dilation (FMD) [8]. Furthermore, a small pilot study showed that moderate carbohydrate intake can improve arterial stiffness and ambulatory blood pressure [9]. However, whether increased carbohydrate intake may alter atherosclerotic progression in patients with established cardiovascular disease remained unclear. In this study, we sought to investigate the relation between dietary macronutrients intake and arterial stiffness in a group of Chinese subjects with established CVD.

## Methods

### Study Populations

This study comprised 364 consecutive patients with established CVD or risk equivalent, recruited from our outpatient medical clinic. Established CVD included prior coronary artery disease and ischemic stroke, while CVD risk equivalence refers to conditions which confer equivalent cardiovascular risk and in our study context included patients with type II diabetes mellitus but without prior CAD/ ischemic stroke. Inclusion criteria constitute a homogeneous sample of subjects with presence of established coronary disease or factors considered to be of equivalent risks (i.e. ischemic stroke or diabetes mellitus) which place subjects at the medium to advanced spectrum of the cardiovascular continuum [7]. Diagnosis of coronary artery disease was established in the presence of any of the following: a history of myocardial infarction; a history suggestive of angina pectoris objectively evidenced by inducible ischemia on exercise treadmill or SPECT; and the presence of coronary atherosclerosis as defined by coronary angiography, computed tomography or magnetic resonance imaging. Diagnosis of ischemic stroke was made on the basis of clinical examinations and computed tomography brain imaging. Patients with type II DM were defined by WHO criteria [10]. Exclusion criteria constituted presence of non-conventional or non-cardiovascular factors which may present important confounding effects that may result in biased estimates of the relations under study or that may cause temporary potential fluctuations in vascular function parameters. These included dilated cardiomyopathy, pregnancy, history of heart failure, significant valvular heart disease, chronic atrial fibrillation, renal failure (serum creatinine > 1.2 mg/dL), cancer, recent myocardial infarction, unstable angina, or coronary revascularization within the past 6 months

During the study period, 93 age- and sex-matched healthy Chinese controls free from history of CVD or diabetes were recruited from a community health screening programme for comparison. The study was approved by the local ethics committee, the Institutional Review Board (IRB) of the University of Hong Kong/ Hospital Authority Hong Kong West Cluster (IRB, HKU/HA HKW Cluster). All participants understood and signed informed consent forms.

### Demographic and clinical assessment

Demographic and clinical data were collected by an interviewer-administered structured questionnaire. Cardiovascular risk factors including tobacco smoking, hypercholesterolemia, hypertension, family history of CVD diagnosed in first-degree relatives before 55 years of age were assessed. Use of cardiovascular medications was recorded. Anthropometric measurements including body weight, height, and waist-hip circumferences ratio were measured. Body mass index (BMI) was then calculated in kg/m [2]. Systolic and diastolic blood pressures were measured. Fasting venous blood sample was obtained from all patients to measure serum glucose, HbA1c, triglyceride, total cholesterol, LDL-C and HDL-C levels.

### Dietary assessment

A validated food frequency questionnaire (FFQ) for Chinese were used to assess dietary intake of all subjects over the past 6 months prior to date of interview, as described previously [11-13]. Briefly, subjects were assessed for frequency (day/week/month/year) and amount (unit of intake: liangs, equivalent to 37.8 g) intake of particular food past year, and 72 major food groups were recorded in FFQ. Types of fruit assessed included apples, pears, tangerines/oranges/grapefruits, bananas, grapes, watermelon, peaches and other fruits. Pictures of food with household units were shown to subjects to increase the accuracy of record. The conversion of dietary nutrients was performed by custom-made software as used in the Shanghai Women’s health Study [12]. High Carbohydrate diet was defined as daily carbohydrate intake of ≥60% of total energy, i.e. above the daily recommended intake (DRI) of carbohydrate at 60% of total energy (Chinese Nutrition Society) [14].

### Arterial Stiffness

Arterial stiffness was measured non-invasively using Vascular Profiling System VP-2000 (Colin Corp., USA). All measurements were performed by a single experienced operator. Patients were allowed to rest in the supine position for 5 minutes in a quiet room before examination. Initially, the sites of maximum carotid and posterior popliteal arteries pulsation were determined by physical examination. Sequential recordings of pressure wave-forms at the precordium and the posterior popliteal arteries were made using hand-held manometer probes with simultaneous electrocardiogram gating. Measurement was taken after achieving coherent reproduction of signals with maximum amplitudes. Heart-ankle pulse wave velocity (PWV) was defined as the distance between the 2 points of measurement over the precordium and the respective posterior popliteal artery, divided by pulse transit time between the systolic R-wave and the upstroke of waveform at the posterior popliteal artery, and was calculated using system software with R-wave synchronisation over at least 10 cardiac cycles. A final estimate was derived from the average of the values from the bilateral measurements. Intra-observer variability testing revealed an intra-class correlation coefficient (two-way mixed, random-effect model, absolute agreement) of 0.87 (95% CI: 0.80 to 0.91, *P < 0.001*) for PWV measurement.

### Statistical Methods

Continuous variables are expressed as mean ± SDs, and categorical data are presented as frequency and percentages. Statistical comparisons were performed by using Student’s t test, Pearson’s χ2 test, or the Fisher’s exact test, as appropriate. Absolute changes and 95% CIs of PWV were calculated by using univariate and multivariate linear regression analysis. Multivariate analyses were performed such that potentially confounding variables and known variables involved in prediction of PWV were chosen, including use of antihypertensive medications and statin [15,16]. Each independent variable was considered a potential confounder if the p-value was <0.20 based on univariate analysis, and was entered and adjusted for in the multivariate Model. Multivariate linear regression analysis was repeated using the overall sample including all subjects, as well as separately among patients with CVD risk equivalent versus healthy controls. In the Full Model, all independent variables were entered and adjusted for all (Table 1). All data analyses were performed using the Statistical Package for the Social Sciences (version 16.0, SPSS). A value of P value less than 0.05 was considered statistically significant.

**Table 1:** Characteristics of the study population and control subjects

	***Study population (n = 364)***	***Control (n = 93)***	***P-value***
***Characteristics***	***Mean (± SD)***	***Mean (± SD)***	
**Age (years)**	66.2 ± 10.2	65.3 ± 7.1	0.417
**Male, *n *** **(%)**	233 (64%)	49 (53%)	0.056
**BMI (kg/m2)**	24.99 ± 3.30	23.80 ± 3.40	0.002*
**Waist-to-hip ratio**	0.93 ± 0.06	0.91 ± 0.08	<0.001*
**Ischemic stroke, *n *** **(%)**	49 (13.46)	----	<0.001*
**Coronary artery disease, *n *** **(%)**	224 (61.54)	----	<0.001*
**Diabetes mellitus, *n *** **(%)**	200 (54.95)	----	<0.001*
**Hypertension, *n *** **(%)**	232 (63.74)	22 (23.66)	<0.001*
**Hyperlipidaemia, *n *** **(%)**	232 (63.74)	30 (32.26)	<0.001*
**Smokers, *n *** **(%)**	165 (45.33)	14 (15.05)	<0.001*
**Systolic blood pressure (mmHg)**	141.00 ± 19.85	127.82 ± 18.04	<0.001*
**Diastolic blood pressure (mmHg)**	79.16 ± 9.19	75.06 ± 9.32	<0.001*
**Total serum cholesterol (mmol/L)**	4.50 ± 0.89	5.12 ± 0.83	<0.001*
**Serum HDL-C (mmol/L)**	1.28 ± 0.34	1.48 ± 0.40	<0.001*
**Serum LDL-C (mmol/L)**	2.56 ± 0.72	3.05 ± 0.76	<0.001*
**Serum triacylglycerol (mmol/L)**	1.49 ± 0.97	1.34 ± 0.74	0.167
**Fasting glucose (mmol/L)**	6.36 ± 2.07	5.16 ± 0.60	<0.001*
**HbA1c (%)**	7.05 ± 1.48	6.63 ± 5.33	0.192
**Medications**			
**Beta-blocker, *n *** **(%)**	184 (50.55)	6 (6.5)	<0.001*
**ACEI/ARB, *n *** **(%)**	36 (9.9)	-----	<0.001*
**Statin, *n *** **(%)**	226 (62.09)	3 (3.2)	<0.001*
**PWV (cm/s)**	1102.17 ± 144.54	1079.48 ***±*** 135.09	0.172

ACEI, angiotensin-converting enzyme inhibitor; ARB, angiotensin receptor blocker; PWV, pulse wave velocity.; HDL-c, high-density lipoprotein cholesterol; LDL-c, low-density lipoprotein cholesterol.

*p-value <0.05.

## Results

### Baseline characteristics

The baseline characteristics of the study population were shown in Table 1. Their mean age was 66.2 ***±*** 10.2 years and 64% of the participants were men. Among them, 62% had documented coronary artery disease, 55% had type II diabetes mellitus and13% had ischemic stroke. Among them, 64% of patients had hypertension and 64% had hyperlipidaemia. And 45% of patients were smokers.

The daily dietary profiles of the study population as measured by FFQ were presented in Table 2. The percentage of energy intake derived from carbohydrate, protein and fat were 72.3 ± 7.6%, 15.2 ± 2.9% and 12.5 ± 5.4%, respectively. Energy intake was significantly reduced among cases compared to controls (*P < 0.001*), with different pattern of intake of major food groups.

**Table 2:** Dietary profile of the study population and control subjects

	***Study population (n = 364)***	***Control subjects (n = 93)***	***P-value***
***Characteristics***	***Mean (± SD)***	***Mean (± SD)***	
**Energy intake/day (Kcal/d)**	1978.22 ± 725.57	2393.10 ± 836.76	<0.001*
**Carbohydrate (g/d)**	359.85 ± 145.80	436.88 ± 168.39	<0.001*
**High carbohydrate diet (≧60%) (n,%)**	346 (95.05)	85 (91.40)	0.207
**Protein (g/d)**	73.95 ± 27.43	87.06 ± 35.26	<0.001*
**Fat (g/d)**	26.97 ± 15.48	32.98 ± 17.70	0.001*
**Carbohydrate (% of energy )**	72.32 ± 7.62	72.54 ± 8.80	0.807
**Protein (% of energy )**	15.21 ± 2.94	14.81 ± 3.68	0.271
**Fat (% of energy )**	12.46 ± 5.39	12.63 ± 5.76	0.794
**Rice**	451.63 ± 191.80	539.36 ± 221.40	<0.001*
**Vegetables**	132.78 ± 98.82	161.07 ± 109.71	0.016*
**Meat**	61.41 ± 58.41	79.25 ± 112.54	0.035*
**Poultry**	19.09 ± 20.85	23.85 ± 25.77	0.062
**Fish**	68.23 ± 70.13	63.56 ± 53.48	0.549
**Egg**	9.25 ± 12.72	9.50 ± 11.00	0.864
**Fruit**	53.95 ± 40.80	70.13 ± 48.98	0.001*
**Soy**	32.79 ± 45.75	49.80 ± 71.00	0.005*
**Fiber**	9.26 ± 6.33	10.63 ± 4.72	0.051
**Saturated fat**	7.00 ± 4.72	9.08 ± 6.43	<0.001*
**Polyunsaturated fatty acids**	5.06 ± 2.63	6.15 ± 3.51	0.002*

*P < 0.05 Significantly different from high carbohydrate diet and without high carbohydrate diet (independent sample *t-*test).

### Effects of dietary high carbohydrate diet on blood pressure and lipid profile

As shown in Table 3, high carbohydrate diet was significantly associated with increased systolic blood pressure [B = 2.86, 95% CI( 1.96 to 3.76), *P < 0.001*], adjusted for age and sex. However, no significant differences were seen in subjects with high carbohydrate diet in terms of diastolic blood pressure, serum LDL-c, HDL-c, and triacylglycerol (*P* > 0.05).

**Table 3:** **Effects of high-carbohydrate diet on blood pressure and serum lipid profile**^**a**^

	***B (95% CI)***	***Adjusted p-value***
**Systolic blood pressure (mmHg)**		
Without high carbohydrate diet (<60%)		
High carbohydrate diet (≧60%)	2.86 (1.96 to 3.76)	<0.001*
**Diastolic blood pressure (mmHg)**		
Without high carbohydrate diet (<60%)		
High carbohydrate (≧60%)	0.68 (-1.26 to 2.62)	0.491
**Total cholesterol (mmol/L)**		
Without high carbohydrate diet (<60%)		
High carbohydrate (≧60%)	23.57 (-38.98 to 86.11)	0.459
**HDL cholesterol (mmol/L)**		
Without high carbohydrate diet (<60%)		
High carbohydrate (≧60%)	-38.96 (-109.85 to 31.93)	0.280
**LDL cholesterol (mmol/L)**		
Without high carbohydrate diet (<60%)		
High carbohydrate (≧60%)	-8.75 (-75.29 to 57.80)	0.796
**Triacylglycerol (mmol/L)**		
Without high carbohydrate diet (<60%)		
High carbohydrate (≧60%)	-2.80 (-28.87 to 23.27)	0.833

^a^Adjusted for age and sex.

*p-value<0.05.

### Effects of high carbohydrate diet on pulse wave velocity

In univariate analysis, high-carbohydrate diet was associated with significantly increased PWV among patients with CVD risk equivalent [B = 72.21, 95% CI (3.80 to 140.62), *P = 0.039*]. Similar association was found among the overall study sample (n = 457) with marginal statistical significance [B = 50.52, 95% CI (-6.03 to 107.07), *P = 0.080*]. Adjusted for potential confounders (including age, sex, diabetes mellitus, smokers, hypertension, hyperlipidaemia, cardiovascular diseases, BMI, and use of medications, including beta-blocker, angiotensin-converting enzyme inhibitor/ angiotensin receptor blocker and statins), dietary energy intake, saturated fat, polyunsaturated fat, monounsaturated fat, fiber, high-carbohydrate diet remained independently associated with PWV [B = 73.50 (10.81 to 136.19), *P = 0.022,* Table 4]. Repeated analysis using Full Model yielded similar results [B = 231.39, 95% CI (87.19 to 375.58), *P = 0.003*]. Nevertheless, no significant association was observed between carbohydrate intake and PWV in healthy controls [B = -28.52, 95% CI (-147.83 to 90.80), P = 0.634, Table 4].

**Table 4:** Unadjusted and adjusted relations of high carbohydrate intake with PWV in study population and control subjects

	**Univariate model**		**Multivariate model**^**1**^					
	***All subjects included (n = 457)***		***All subjects included (n = 457)***		***CVD/ risk equivalent (n = 364)***		***Healthy controls (n = 93)***	
	**β *(95% CI)***	***p-value***	**β *(95% CI)***	***p-value***	**β *(95% CI)***	***p-value***	**β *(95% CI)***	***p-value***
**Age**	6.61 (5.39 to 7.83)	<0.001*	6.68 (5.42 to 7.94)	<0.001*	6.27 (4.93 to 7.61)	<0.001*	9.88 (5.77 to 13.99)	<0.001*
**Male**	28.08 (1.17 to 55.00)	0.041	33.38 (8.40 to 58.36)	0.009*	23.55 (-4.46 to 51.56)	0.099	67.87 (10.17 to 125.58)	0.022*
**Diabetes mellitus**	18.81 (-8.19 to 45.80)	0.172	46.34 (18.93 to 73.76)	0.001*	43.18 (15.22 to 71.14)	0.003*	150.75 (-88.20 to 389.71)	0.212
**Hypertension**	69.97 (44.07 to 95.87)	<0.001*	53.52 (27.82 to 79.21)	<0.001*	58.61 (30.39 to 86.83)	<0.001*	22.69 (-45.87 to 91.25)	0.511
**Hyperlipidaemia**	13.60 (-13.88 to 41.08)	0.331						
**Past or current smokers**	49.39 (-67.71 to 166.48)	0.401						
**BMI**	-1.14 (-5.07 to 2.79)	0.569						
**Medications**								
**Beta-blocker**	22.17 (-5.02 to 49.36)	0.110	-0.90 (-27.55 to 25.75)	0.95	0.29 (-27.35 to 27.92)	0.984	-17.61 (-130.81 to 95.60)	0.757
**ACEI/ARB**	19.31 (-29.41 to 68.03)	0.436						
**Statin**	6.17 (-20.43 to 32.76)	0.649						
**Dietary energy intake**	0.003 (-0.015 to 0.020)	0.769						
**Saturated fatty acid**	-2.31 (-4.84 to 0.22)	0.074	-0.58 (-3.03 to 1.87)	0.643	0.47 (-2.42 to 3.35)	0.750	-3.39 (-8.07 to 1.28)	0.151
**PUFA**	-2.12 (-6.71 to 2.47)	0.364						
**MUFA**	-4.97 (-2.33 to 1.33)	0.594						
**Fiber**	-0.103 (-2.28 to 2.07)	0.926						
**CVD/risk equivalent**^**3**^	22.68 (-9.90 to 55.26)	0.172	-51.81 (-90.32 to -13.30)	0.008*	-	-	-	-
**High carbohydrate diet (≧60%)**	50.52 (-6.03 to 107.07)	0.080	52.46 (-3.09 to 108.01)	0.064	73.50 (10.81 to 136.19) Full Model Estimate^2^: 231.39 (87.19 to 375.58)	0.022* 0.003*	-28.52 (-147.83 to 90.80)	0.634

CI, confidence interval; CVD, cardiovascular disease; BMI, body-mass index; ACEI, angiotensin-converting enzyme inhibitor; ARB, angiotensin receptor blocker; PUFA, polyunsaturated fatty acid; MUFA monounsaturated fatty acid; PWV, pulse wave velocity.

^1^Multivariate Model, adjusted for potential confounders with p-value <0.20 based on univariate analysis.

^2^Full Model – Entered and adjusted for all independent variables.

^3^CVD/Risk Equivalent – included coronary artery disease, ischemic stroke, and type II diabetes mellitus without prior coronary artery disease/ischemic stroke.

*p-value<0.05.

## Discussion

High-carbohydrate diet is associated with increased cardiovascular risk [17-19], in terms of adverse changes in cardiovascular risk factors, such as raised serum triglycerides and reduced levels of high-density lipoprotein cholesterol, but its relation with vascular function remained unclear. In coherence with an earlier small pilot study showing that moderate carbohydrate diet reduced PWV [9], this study further shows that high-carbohydrate diet is independently associated with increased PWV in patients with established cardiovascular disease or risk equivalent. Arterial stiffness as measured by PWV has been shown to be an important clinical marker of atherosclerosis [20,21], and is a predictor of cardiovascular events in healthy subjects beyond traditional risk factors [22]. The results of this study suggest that increased carbohydrate intake in patients with established cardiovascular disease or risk equivalent may potentially contribute to further increased risk of future cardiovascular events through accelerated progression of atherosclerosis. The mechanisms linking arterial stiffness to CVD are not completely understood, and may involve insulin resistance promoting endothelial dysfunction, oxidative stress, vascular smooth muscle cell growth and stimulation of the sympathetic nervous system [23].

There are several possible mechanistic pathways through which high-carbohydrate diet could impact on atherosclerosis, and these included changes in blood pressure, lipid profile, or modulation of inflammatory cytokines and the nitric oxide system [24,25]. In this study, increased carbohydrate intake is associated with raised systolic blood pressure, which is consistent with the previous finding of beneficial changes in ambulatory blood pressure associated with a low glycemic index diet [9]. No changes were observed in glycemic and lipid profiles in this study in relation to a high carbohydrate diet. These findings suggest that high-carbohydrate diet may affect arterial stiffness through altering BP but not lipid profile.

Notably, the percentage of smokers among patients with established cardiovascular diseases was markedly higher than that among control subjects (45.3% versus 15.1%, *P < 0.001*). The adverse effects of cigarette smoke in atherogenesis are indispensible, mediated by multiple pathways including free radical-induced oxidative stress as well as pro-thrombotic and pro-inflammatory cascades [26]. We therefore carefully adjusted for the effects of smoking in the multivariate analysis, and showed that the pro-atherosclerotic effects of high carbohydrate diet were independent of smoking. Interestingly, the pro-atherosclerotic effect of high carbohydrate diet was present in patients with cardiovascular disease or risk equivalent, but not in healthy control subjects. This suggests that high carbohydrate diet may preferentially affect the most susceptible patients at the advanced spectrum of the cardiovascular continuum. This implies that dietary interventions [27] to reduce habitual carbohydrate intake may have an important role in secondary prevention of cardiovascular disease.

### Study limitations

First, the glycemic index was not determined in this study, therefore direct comparison with previous studies could be difficult and the specific type of carbohydrate cannot be ascertained. Second, a direct causality of dietary carbohydrate intake and arterial stiffness cannot be established in a cross-sectional setting. Furthermore, the method in measuring heart-ankle PWV was not *a priori* validated in our study. Also, the small sample size of the patient sample (n = 364) and the control sample (n = 93), and the small number of subjects with low carbohydrate intake, also limits the conclusions draw from the multivariate analysis as well as the statistical power of the study. Future large randomized controlled studies will be important to confirm the causality by investigating the potentially protective effects of reduced carbohydrate intake against atherosclerotic progression in patients with high risk of cardiovascular events.

## Conclusions

High habitual carbohydrate intake is associated with increased arterial stiffness in patients with established cardiovascular disease or risk equivalent.

## Competing interests

The authors declare that they have no competing interests.

## Authors’ contributions

HTC performed the data collection and analysis, drafted the manuscript, and revised it critically for important intellectual content; YHC, KHY, and CPL participated in study design and execution, drafted the manuscript and revised it critically for important intellectual content; SWL participated in study design, data collection and site management; ST performed the biochemical measurements; HFT supervised the overall vascular function study design and direction, data collection and critical analysis, and writing/revision of manuscript. All authors read and approved the final manuscript.

## Pre-publication history

The pre-publication history for this paper can be accessed here:

http://www.biomedcentral.com/1471-2261/14/24/prepub
